# RLetters: A Web-Based Application for Text Analysis of Journal Articles

**DOI:** 10.1371/journal.pone.0146004

**Published:** 2016-01-05

**Authors:** Charles H. Pence

**Affiliations:** Department of Philosophy and Religious Studies, Louisiana State University, Baton Rouge, Louisiana, United States of America; Virginia Tech, UNITED STATES

## Abstract

While textual analysis of the journal literature is a burgeoning field, there is still a profound lack of user-friendly software for accomplishing this task. RLetters is a free, open-source web application which provides researchers with an environment in which they can select sets of journal articles and analyze them with cutting-edge textual analysis tools. RLetters allows users without prior expertise in textual analysis to analyze word frequency, collocations, cooccurrences, term networks, and more. It is implemented in Ruby and scripts are provided to automate deployment.

## Introduction

The journal literature is massive, and grows at an astounding rate. One estimate, for example, puts the size of the Google Scholar index at around 160 million documents [1]. Depending on the database and measurement method utilized, estimates for its annual growth rate vary, but one research team offers values ranging from 2.2% to 9% [2]. If the higher numbers are right, then more than ten million articles are published every year, and the entire literature will double in just under a decade. This fact has a variety of implications. First and foremost, it is and will remain exceptionally difficult for individual researchers to keep pace with the rate of publication, even within their own specialties [3]. Equally troubling, it is exceptionally difficult to perform meta-level studies of the journal literature. If we wish to consult a statistically significant, broad, longitudinal cross-section of publications, it is more and more evident that we simply cannot read enough articles.

With the digitization of the journal literature, however, a new way of answering such questions is beginning to emerge. A substantial fraction of the journal literature has now been digitized, numbering in the hundreds of millions of pages. This opens up opportunities to use automated tools to examine at a broad scale how research happens. Unfortunately, however, there is a lack of tools for studying this corpus of texts in a rigorous yet user-friendly way.

In order to address the lack of tools for automated analysis of the journal literature, we have created RLetters, a general-purpose, web-based tool optimized for the analysis of journal articles in plain text. RLetters may be deployed by any user desiring to host their own searchable and analyzable archive of journal articles, and requires only a web server running Ruby on Rails and an Apache Solr server, each of which is relatively simple to deploy. RLetters comes with scripts which can automate the process of deployment to a new server or virtual machine. A JSON-based API for search is already available, and further interoperability is planned for future versions.

When compared to extant tools for textual analysis, we believe that RLetters offers advantages over most of the solutions now available. Several, including TAPoR Tools [4] and MONK [5], require that the user upload texts into the system, making it prohibitive to study a large corpus. Some, such as MONK, require for full capability that the texts be marked up manually in a format like TEI [6], which, again, is impossible for analysis of a large corpus. Journal article analyses must be performed using unadorned plain text, as this is often the only format available from publishers. Other tools, such as Google’s N-grams viewer [7] and JSTOR’s Data for Research [8], have fixed corpora of text against which they are deployed, and thus cannot be readily applied to a user’s preferred area of research. No general-purpose tool presently available is optimized for journal articles—the challenges presented by the analysis of millions of small texts (rather than a much smaller number of much larger texts) are unique and significant. Finally, some current programs (such as SEASR [9] or TAPoR Tools) require the user to chain together many smaller analysis steps to perform common data analyses, presenting a usability challenge. RLetters resolves each of these concerns.

## Text Analysis Methods

A wide variety of analysis methods are implemented in RLetters. The more common analyses are described quickly, and two of RLetters’s more powerful algorithms are discussed in more detail.

It is advised, as with all digital humanities tools, that users interested in working with these analysis methods for publishable work not treat RLetters as a black box. Each of the analysis methods available has its limitations and its advantages, and some varieties of data for which it is suitable and some for which it is not. Users should locate papers or books describing each method (some of which are provided as citations in the following) and investigate these to determine whether or not their analyses provide the sort of insights they require.

### Common Analysis Methods

#### Compute Term Frequency Information

Users can compute term frequency tables for a given dataset, for either single words or multiple-word phrases (n-grams). These are the most common inputs for other kinds of textual analysis algorithms, meaning that users can easily extract term frequencies and use them to run their own analyses locally if desired. The options and features in the word frequency generator are modeled after those found in KWIC Concordance [10], modified and expanded to accommodate the needs of some of the other analysis algorithms.

#### Compute Term Network

Users can visualize the network of words occurring in the immediate vicinity of a given focal word of interest, an analysis useful for determining which words often “travel together” in the literature [11].

#### Extract Proper Names

All proper names (of persons, locations, organizations, and so forth) found in journal articles can be extracted. This analysis can be useful to detect locations of field research, organizational networks, etc. This analysis uses the Stanford Natural Language Processing toolkit [12].

#### Graph by Publication Date

Users can graph the publication dates of a dataset, which is particularly useful if the dataset contains only those articles which match a complex search.

#### Export Citations

Lastly, a dataset can be exported in a variety of citation formats to a user’s citation manager, including EndNote and BibTeX.

### Craig Zeta: Compare Word Usage in Two Datasets

Users can request an analysis of words which are likely to mark out an article’s belonging to one dataset as opposed to another. The algorithm implemented here is the Zeta algorithm, as described originally by Burrows [13] and extended by Craig and Kinney [14]. This algorithm is aimed at the determination of *difference*. Consider some corpus, divided into two groups, A and B. The Zeta algorithm tells us which words within A mark a text out as (probably) belonging to A rather than B (and vice versa). Further, the Zeta algorithm has the advantage of providing us with words that are far less likely to appear overall, as opposed to words singled out by traditional T-tests. For example, while a T-test might pick out the more frequent use of a common word like ‘upon’ as a marker of membership in one group rather than another, the Zeta algorithm is more likely to give us much rarer, and hence more meaningful words.

The Zeta algorithm is relatively simple to describe. We take our two groups of texts (A and B) and divide them into chunks of 500 words. For each word *w* in the corpus, we then simply add the fraction of A chunks in which *w* appears and the fraction of B chunks in which *w* does *not* appear. The maximal Zeta score for a word is thus 2—for words that appear in every chunk of A and in no chunk of B. The minimal score would be 0—for words that appear in no chunk of A and in every chunk of B. Clearly, high-scoring words are a very good indicator of a text’s belonging to the A group, and low-scoring words are a very good indicator of membership in the B group.

The output of the algorithm, therefore, is two collections of “marker words”—words that, if found in a given random sample of text, mark that text out as, respectively, much more likely to be a member of dataset A or a member of dataset B. For an example of how this algorithm can be used in practice, see below.

### Cooccurrence and Collocation Analysis

Users can use RLetters to locate both statistically significant immediate pairs of words (known as collocations), or to detect significant correlations between words at the sentence, paragraph, or article level (known as cooccurrences) [15].

Collocation analysis is commonly used to track technical terms or immediate patterns of word usage. To refer to a common example from linguistics, collocation analysis can detect that “strong tea” is a common locution in English, and that “powerful computers” is as well, but that both “powerful tea” and “strong computers” would be highly unusual, as these two pairs are very rarely found in a standard English corpus. Collocation analysis can also detect technical term usage, such as the strong association of word pairs like “genetic drift” or “statistical mechanics.”

Cooccurrence analysis extends collocation analysis to look for significant correlations within sections of a document of a given size. (Common sizes include 25 words, roughly corresponding to sentence-level cooccurrences, 100 words [paragraph-level], 500 words [section-level], and entire articles.) The significance of cooccurrences can be measured using a variety of traditional statistical measures, including mutual information, T-tests, or log-likelihood. Cooccurrences provide a different sort of information about texts than collocations. Sentence- and paragraph-level cooccurrence provides interesting information about patterns of speech—for instance, that researchers working on human decision-making very often speak of “recognition” and “inference” in the same sentence, but only very rarely connect “recognition” and “aversion” (drawn from an unpublished study in progress).

## Implementation

The backend of RLetters consists of an Apache Solr server [16], used both as a search engine and, effectively, as a single-table database. This server contains document records consisting of metadata about each document (citation data, license information, etc.), and a detailed index of the full text of each article.

**Fig 1:**
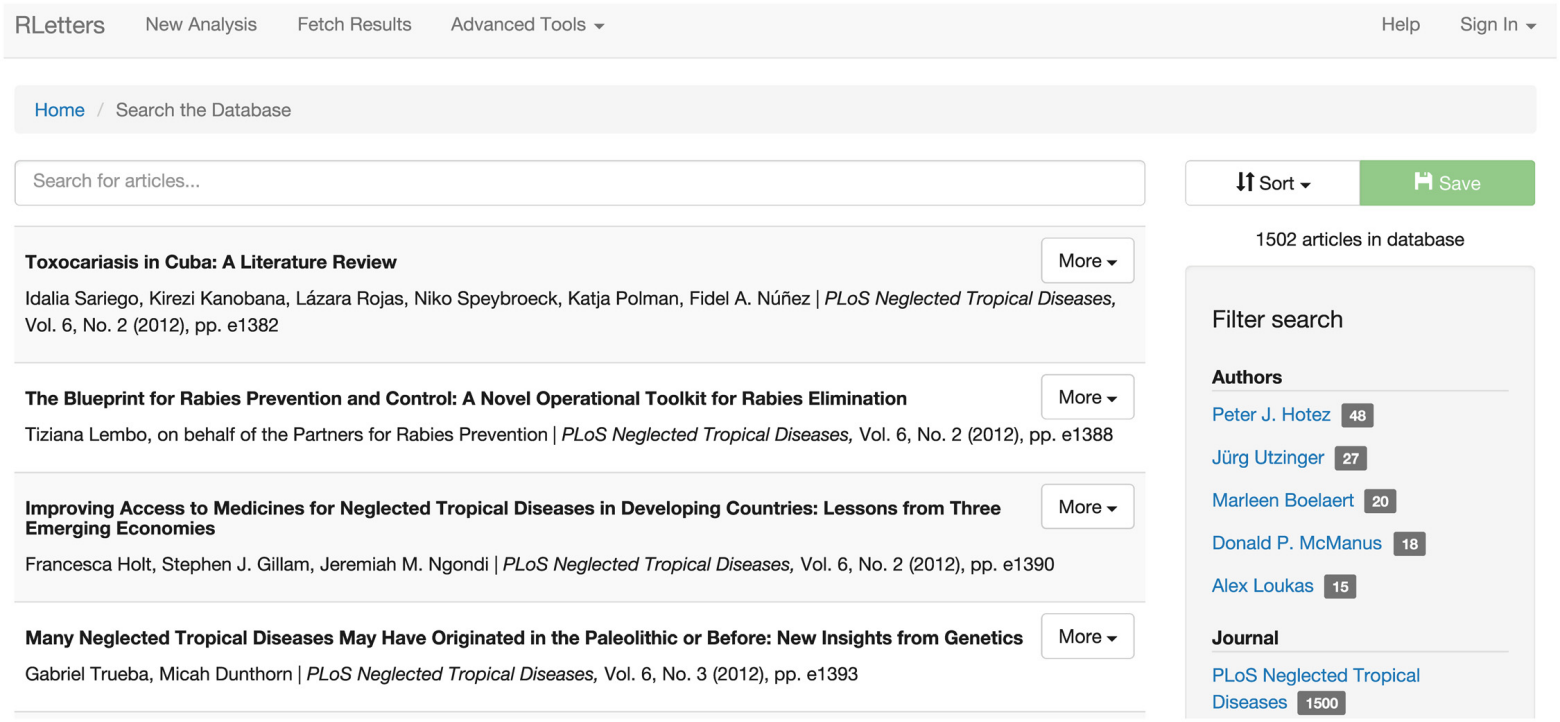
The basic search interface for RLetters. In this screenshot of RLetters’ test database, containing the entirety of *PLoS Neglected Tropical Diseases*, we see an example page of search results, showing links to filter the data and retrieve more information about each article.

The frontend is implemented as a web application in Ruby on Rails. The web application first proceeds by allowing the user to select a research question in which he or she is interested. The next step is to provide data for the analysis—a set of articles of interest, saved as a permanent object called a ‘dataset’. Datasets are constructed by performing complicated searches using the search interface, which allows users to search on a variety of different metadata fields (including author, title, journal, licensing information, etc.) and filter the results by author, journal, and publication date. Once the desired set of articles has been located, it is named and saved for future use in analyses. See Fig 1 for a screenshot of RLetters’ search interface.

The dataset is then analyzed using the algorithm appropriate to answering the intended question. Data analyses in RLetters are not interactive, as some are quite computationally expensive. Analysis is thus executed in the background and, upon completion, users are e-mailed and return to the site to retrieve their results. While it is difficult to provide accurate timing for how long a given job will take, users are shown a list of estimated completion times for datasets of various sizes, and can use these as a rough idea of how long their analysis might take to complete. All results produced in RLetters may be viewed online as well as exported, both in open formats (such as CSV spreadsheets) and, in many cases, as visualizations (such as PDF files).

The source code of RLetters can be freely downloaded from its website, http://www.rletters.net, or at its GitHub page, http://github.com/rletters/rletters. It is released as open-source software under the MIT License, and is under active development. Further, it comes with a set of scripts which automate the deployment of an RLetters instance to any RHEL 7 or CentOS 7 server.

A demonstration instance of RLetters may be found at https://demo.rletters.net, containing a variety of full-text articles from various *PLoS* journals. The demonstration database contains 13,555 articles and the full suite of RLetters analysis tools.

### Input Data and Copyright

As with most textual analysis endeavors, the hardest problem with performing large-scale analysis of the journal literature is obtaining rights to the full text of journal articles. RLetters takes as its input a set of XML files containing the full text of articles as well as metadata about them, including authors, title, journal, DOI, and licensing information. With Open Access articles, such as those published in the *PLoS* journals or those available as part of the PubMed Central Open Access Subset [17], simple transformations from the freely available NLM XML/JATS format [18] allow for rapid data input into RLetters (see the collection of scripts at http://github.com/cpence/evotext).

For closed access content, RLetters has the ability to store only the metadata for an article and a link to its full text as stored on an external server. When an analysis of a closed-access article is requested that requires the full plain text of an article, it is fetched, analyzed, and destroyed when the analysis completes. Future versions of RLetters will include support for interfacing with the text-mining APIs of journal publishers, such as that provided by Elsevier [19].

## Example

To demonstrate the sort of analysis that RLetters can perform, an example analysis was run using the RLetters Demo, available publicly at https://demo.rletters.net. As mentioned above, this installation contains some thirteen thousand articles from *PLoS* journals, taken from their freely available NLM XML-format full text. These data are available under the CC-BY license. The server was run using the latest version of RLetters (v2.0.0).

Our example considers the following question. Imagine a researcher with a moderately-computational article, attempting to decide whether to submit his or her article to *PLoS Biology* or *PLoS Computational Biology*. The publication guidelines for *PLoS Computational Biology* state that it seeks manuscripts which “further our understanding of living systems at all scales—from molecules and cells, to patient populations and ecosystems –through the application of computational methods.” But how might a researcher be more certain that his or her article is “computational enough” to qualify? Here, the Zeta algorithm can help. Applying Zeta to the corpora of *PLoS Computational Biology* and *PLoS Biology* can give us lists of marker words—and if a sufficient number of the *Computational Biology* marker words appear in our researcher’s paper, he or she can make a much more educated guess about the appropriate home for the manuscript in question.

**Fig 2:**
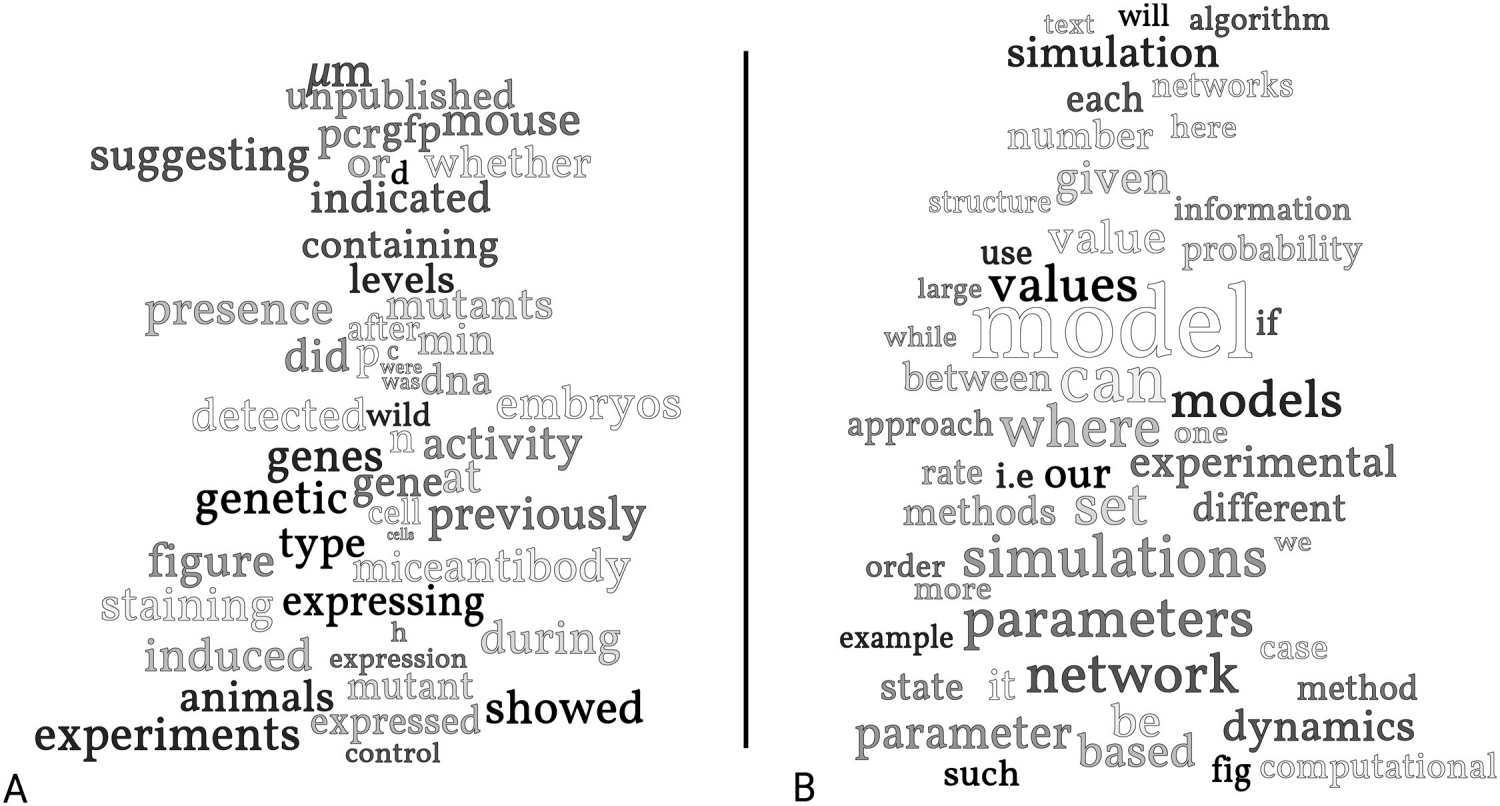
Marker words for *PLoS Biology* vs. *PLoS Computational Biology*. The fifty words (scaled according to strength of inference) that let us infer that a randomly selected manuscript likely belongs in *PLoS Biology* rather than in *PLoS Computational Biology* (A) and vice versa (B). Word clouds generated by RLetters.

To get these sets of marker words, two datasets were constructed—one containing all articles from *PLoS Biology* and one containing all articles from *PLoS Computational Biology*. The marker lists were generated, and RLetters also created word clouds for each set of marker words, included as Fig 2A and 2B. If a random article contains words from the right-hand word cloud, that is, it is likely to be within the scope of *PLoS Computational Biology*, while if it contains words in the left-hand cloud, it is likely to belong better at *PLoS Biology*.

The marker words show a few expected trends, and a few surprises. On the unsurprising side, *Computational Biology* papers are more likely to discuss “models,” “simulations,” “parameters,” “value(s),” “networks,” and “dynamics.” *Biology* papers, on the other hand, are more likely to discuss “animals,” “mice,” “genes,” and the details of experimental protocol, such as “staining,” “antibodies,” “PCR,” “GFP,” and tissue sections measured in “*μm*.”

More interestingly, however, we see changing patterns of usage in the way that experiments themselves and their results are discussed. In *Biology* articles, for example, “experiments” are discussed, while in *Computational Biology* articles, “experimental” is the common usage. A follow-up collocation analysis indicates that some of the most frequent pairs including “experimental” are “experimental data,” “results,” “observations,” “evidence,” and “studies.” Further, *Biology* articles are more tentative about their results—the words “suggesting,” “indicated,” and “whether” are all markers for *PLoS Biology*.

This example, therefore, points to more than a simple difference in focus between *PLoS Biology* and *PLoS Computational Biology*. The very language used to discuss the experimental data that these articles invoke is profoundly different. Consulting these lists of marker words should, in fact, make it very clear which disciplinary idiom a given article uses.

## Conclusion

There is a demonstrated need for usable applications in text analysis of the journal literature, and RLetters helps to fill this need. We provide here a full-featured web application that can be deployed by anyone wishing to analyze a corpus of journal articles. Our example demonstrates that even in relatively straightforward applications, this level of textual analysis can offer interesting and useful insights into the journal literature that provide tangible benefits to researchers.
